# Influence of La and Ce Additions on the Microstructure and Stress Rupture Property in GH4079 Superalloy

**DOI:** 10.3390/ma19163535

**Published:** 2026-08-20

**Authors:** Ping Yu, Xiangyi Hou, Dong Liu, Yinjun Feng, Qiang Tian, Xintong Lian

**Affiliations:** 1 CISRI-GAONA Co., Ltd., Beijing 100081, China; 18401643815@163.com; 2 State Key Laboratory of Materials for Advanced Nuclear Energy, Shanghai University, Shanghai 200444, China; houxiangyi@shu.edu.cn (X.H.); fyj_mse@163.com (Y.F.); 3 AECC Guizhou Liyang Aviation Power Co., Ltd., Guiyang 550014, China; liudcb@126.com

**Keywords:** Ni-based superalloy, rare earth elements, precipitation, stress rupture property

## Abstract

GH4079 is a newly developed Ni-based superalloy with a high content of alloying elements and excellent high-temperature mechanical performance. Rare earth (RE) elements are always added to superalloys to improve their stress rupture properties. This study explored how varying additions (0–0.22 wt.%) of lanthanum (La) and cerium (Ce) affected the precipitation and stress rupture performance of the GH4079 superalloy and revealed the related strengthening mechanisms. The addition of RE elements restrained the excessive growth of the γ′ phase to keep its particles fine and uniform, while more RE led to a larger quantity and size of RE-rich precipitates and carbides. At 650 °C and 882 MPa, La and Ce notably boosted the alloy’s stress rupture life. The sample with 0.22 wt.% RE showed optimal overall performance, with a stress rupture life of 179.3 h, more than three times that of the RE-free alloy (55.2 h). The improvement in the stress rupture properties by La and Ce was attributed to beneficial intracrystalline and grain boundary effects. RE reinforced the γ′ phase to impede dislocations and purified the grain boundaries by decreasing the segregation of P and S elements. Additionally, small-sized RE-rich precipitates combined with the fine γ′ phase formed zigzag grain boundaries, alleviating stress concentration and retarding intergranular crack propagation.

## 1. Introduction

The GH4079 superalloy is a precipitation-strengthened deformation superalloy with a high volume fraction (45%) of the γ′ phase based on the Ni-Co-Cr alloy system [1,2]. It is mainly used for aero-engine turbine disks working at high temperatures due to its excellent strength and corrosion resistance [3,4]. After the alloy is forged and solid solution-treated, the primary γ′ phase is completely dissolved, and a fine secondary γ′ phase is precipitated during cooling, which improves the high-temperature strength and structural stability [5,6,7]. However, the stress rupture life tested under 650 °C/882 MPa conditions of some domestically produced GH4079 alloy forgings shows obvious deficiencies or fluctuations, making it difficult to stably meet the technical requirements of greater than 100 h. As the operating environment of aero-engines becomes increasingly severe, higher requirements are placed on the comprehensive properties of GH4079 alloy, and optimizing its microstructure and improving its stress rupture life have become an important research focus.

Rare earth (RE) elements are well-known microalloying elements in the metallurgical field, which can often purify grain boundaries and optimize precipitation behavior, thus improving the high-temperature mechanical properties of alloys [8,9,10,11]. Tian et al. [12] showed that RE and B synergistically enhanced stress rupture life by increasing the binding force of the grain boundaries, purifying boundaries, and inhibiting carbide precipitation. Silveira et al. [13] showed that yttrium additions (0.011–0.067 wt.%) to alloy 718 increased the creep rupture time and reduced the minimum creep rate by suppressing grain boundary sliding and lowering the stacking fault energy. Hu et al. [14] demonstrated that RE elements, synergistically with P, improved stress rupture properties by co-segregating at grain boundaries to form RE phosphides, which enhanced grain boundary stability and hindered intergranular cracking. Previous studies have revealed that the addition of RE elements can improve the stress rupture life of superalloys, and a small variation in RE content can even cause a significant fluctuation in stress rupture life [15,16,17,18]. This indicates that RE elements have a highly sensitive regulatory effect on the high-temperature endurance performance of superalloys. The dominant mechanisms by which RE elements improve endurance performance mainly include grain boundary purification, modification of inclusions, and the regulation of grain boundary precipitated phases. Based on the widely reported beneficial effects of lanthanum (La) and cerium (Ce) individually in superalloys, it is expected that their addition will play a significant role in optimizing the microstructure and improving the stress rupture life of the GH4079 alloy [19,20,21]. Furthermore, boron is a widely used trace element in superalloys. Previous studies have shown that adding boron to nickel-based superalloys can effectively improve their stress-rupture and creep properties [22,23]. Meanwhile, synergistic effects between boron and rare earth elements have been reported, where their combined addition strengthens grain boundaries and leads to a superior stress-rupture life compared to adding either element alone [12]. The GH4079 alloy inherently contains a certain amount of boron (<0.01 wt.%). Based on this intrinsic compositional characteristic, RE elements (La and Ce) were added in this study, aiming to achieve further property improvement beyond the beneficial effects of boron.

The γ′ phase is the primary strengthening phase in the GH4079 alloy. Its size and volume fraction are key factors influencing the mechanical properties of nickel-based superalloys [24,25]. It impedes dislocation motion via shearing or the Orowan looping mechanism, depending on which mechanism requires a lower critical resolved shear stress [26]. RE elements may affect the size and lattice misfit of γ′ precipitates, thereby modifying precipitation strengthening and consequently influencing dislocation behavior [27].

At present, there are few systematic studies on the effect of RE addition on the microstructure and stress rupture properties of the GH4079 superalloy, and the mechanism of its effect on grain-boundary structure and dislocation movement still needs further verification. In this paper, by adding different RE contents of La and Ce to the GH4079 superalloy, the effects of RE elements on the microstructure and stress rupture properties of the alloy at 650 °C/882 MPa were studied, with the aim of addressing the insufficient and fluctuating stress rupture life observed in some GH4079 forgings. Based on grain size, the γ′ phase, grain-boundary precipitated phases, inclusions, and fracture characteristics, the strengthening mechanisms of RE elements in the alloy were explored and analyzed by means of advanced characterization methods such as SIMS and TEM. Due to practical production costs and process considerations, La and Ce were added simultaneously, which limits the ability to distinguish the individual effects of each RE element. The separate influence of La and Ce on the alloy requires further investigation in the future. This work is expected to provide a theoretical reference for the performance optimization of the GH4079 superalloy and has important engineering significance for extending the service life of aero-engine turbine disks and ensuring operational safety.

## 2. Experimental

### 2.1. Materials and Experimental Procedure

Four GH4079-based alloys with different RE contents were designed. The La/Ce mass fractions were 0/0, 0.025/0.005, 0.050/0.010, and 0.150/0.070 wt.%. To facilitate subsequent analysis, the four test alloys were designated as 0RE, 0.03RE, 0.06RE, and 0.22RE respectively. The La/Ce mass ratio was initially designed to be 5:1 for all RE-containing alloys. However, due to the higher chemical activity of La compared with Ce, La suffered greater melting loss through oxidation and slagging during vacuum melting, especially at high RE addition levels. As a result, the actual La/Ce ratio in the high-RE alloy decreased to approximately 2:1, while the designed 5:1 ratio was well retained in the low-RE alloys. The specific composition design is shown in Table 1. As their designed RE contents (La/Ce) exceed the limits specified for standard GH4079 in GB/T 14992-2025 [28], they are described as GH4079-based alloys. The minor variations in other alloying elements, such as Al, Cr, Fe, and Nb across these alloys are within the compositional tolerance specified in GB/T 14992-2025. 

The test alloy ingots were prepared by a dual process of vacuum induction melting and electroslag remelting. After the ingots underwent homogenization treatment at 1160 °C and were hot-extruded into Φ80 mm bars at 1160 °C, they were then subjected to a four-step heat treatment including annealing at 1040 °C for 6 h, solution treatment at 1140 °C for 6 h, aging at 850 °C for 6 h, and aging at 780 °C for 12 h.

### 2.2. Microstructure Characterization

Metallographic specimens of 10 × 10 × 10 mm were ground, polished, and then chemically etched in a solution consisting of 100 mL of hydrochloric acid, 100 mL of ethanol, and 20 g of copper chloride. An Olympus GX71 (Olympus Corporation, Tokyo, Japan) optical microscope (OM) and Gemini SEM 300 (Carl Zeiss Microscopy GmbH, Oberkochen, Germany) field-emission scanning electron microscope (SEM) were used to observe the microstructure of the samples. Grain size and precipitate size were measured using Image-Pro Plus 6.0 software. The fracture surfaces of the stress rupture samples were cleaned in an acetone solution for 15 min and then observed by SEM. An FEI Talos F200X (FEI, Waltham, MA, USA) transmission electron microscope (TEM) was employed to characterize the distribution of precipitated phases and the dislocation configurations. TEM samples were prepared by grinding to a thickness of less than 50 µm, punching into 3 mm disks, and then electrolytically thinning at −20 °C and 24 V in a solution of 90% ethanol (C_2_H_5_OH) + 10% perchloric acid (HClO_4_). Elemental segregation was analyzed using a TOF-SIMS 5-100 (IONTOF GmbH, Münster, Germany) time-of-flight secondary ion mass spectrometer (TOF-SIMS) at 30 keV with a 45 degree incident angle, focusing on a region of 50 × 50 μm.

### 2.3. Stress Rupture Test

**Figure 1:**
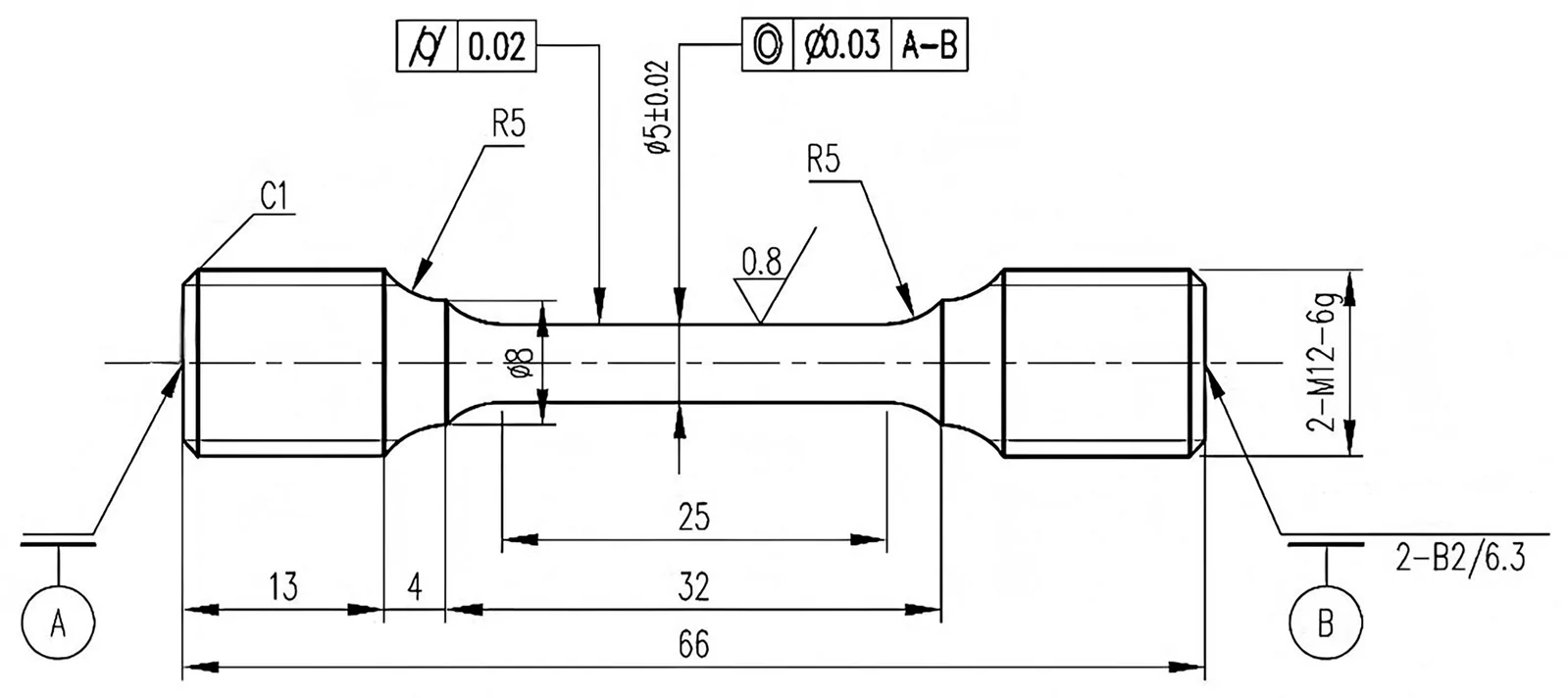
Stress rupture samples [30].

Test samples were taken from the same location in the forged bar and machined into stress rupture test specimens. Two parallel specimens from each group with different RE contents were taken for stress rupture testing, and the average values were calculated. The dimensions of the specimens are shown in Figure 1. Stress rupture tests were conducted at 650 °C/882 MPa according to the GB/T 2039-2024 [29] standard.

## 3. Results

### 3.1. Initial Microstructure

**Figure 2:**
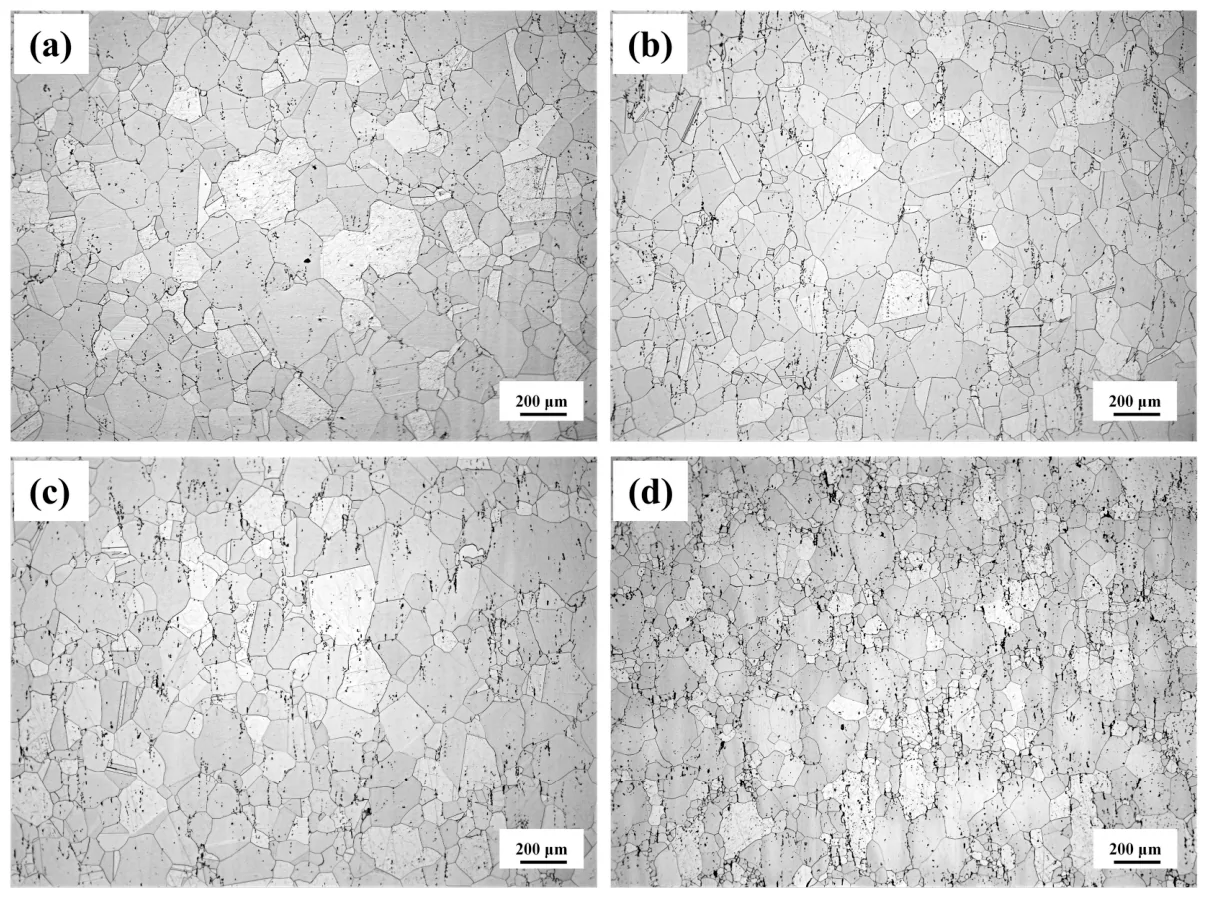
Microstructure in different GH4079-based test alloys: (**a**) 0RE alloy; (**b**) 0.03RE alloy; (**c**) 0.06RE alloy; (**d**) 0.22RE alloy.

Figure 2 shows the microstructures of the different GH4079-based test alloys. The average grain size of the 0RE alloy was 175 μm, and that of the 0.03RE alloy was 160 μm. As the RE content continued to increase, the grain size kept decreasing from 155 μm in the 0.06RE alloy to 121 μm in the 0.22RE alloy. Meanwhile, the increase in RE content also led to an increase in the number of grain boundary precipitates, which pinned the grain boundaries. However, due to the uneven distribution of the precipitates, a mixed-grain structure was observed. 

**Figure 3:**
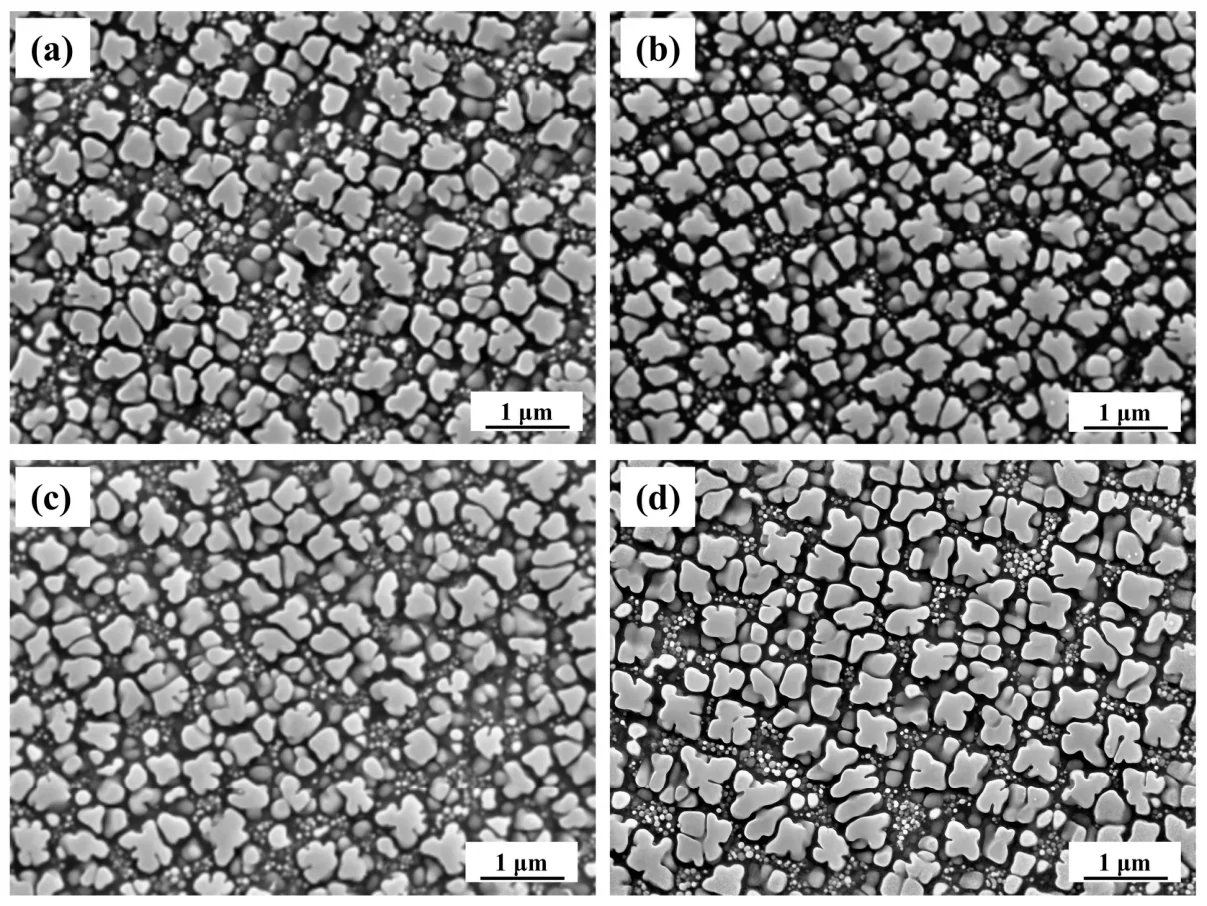
Morphology of γ′ phases in different GH4079-based test alloys: (**a**) 0RE alloy; (**b**) 0.03RE alloy; (**c**) 0.06RE alloy; (**d**) 0.22RE alloy.

**Figure 4:**
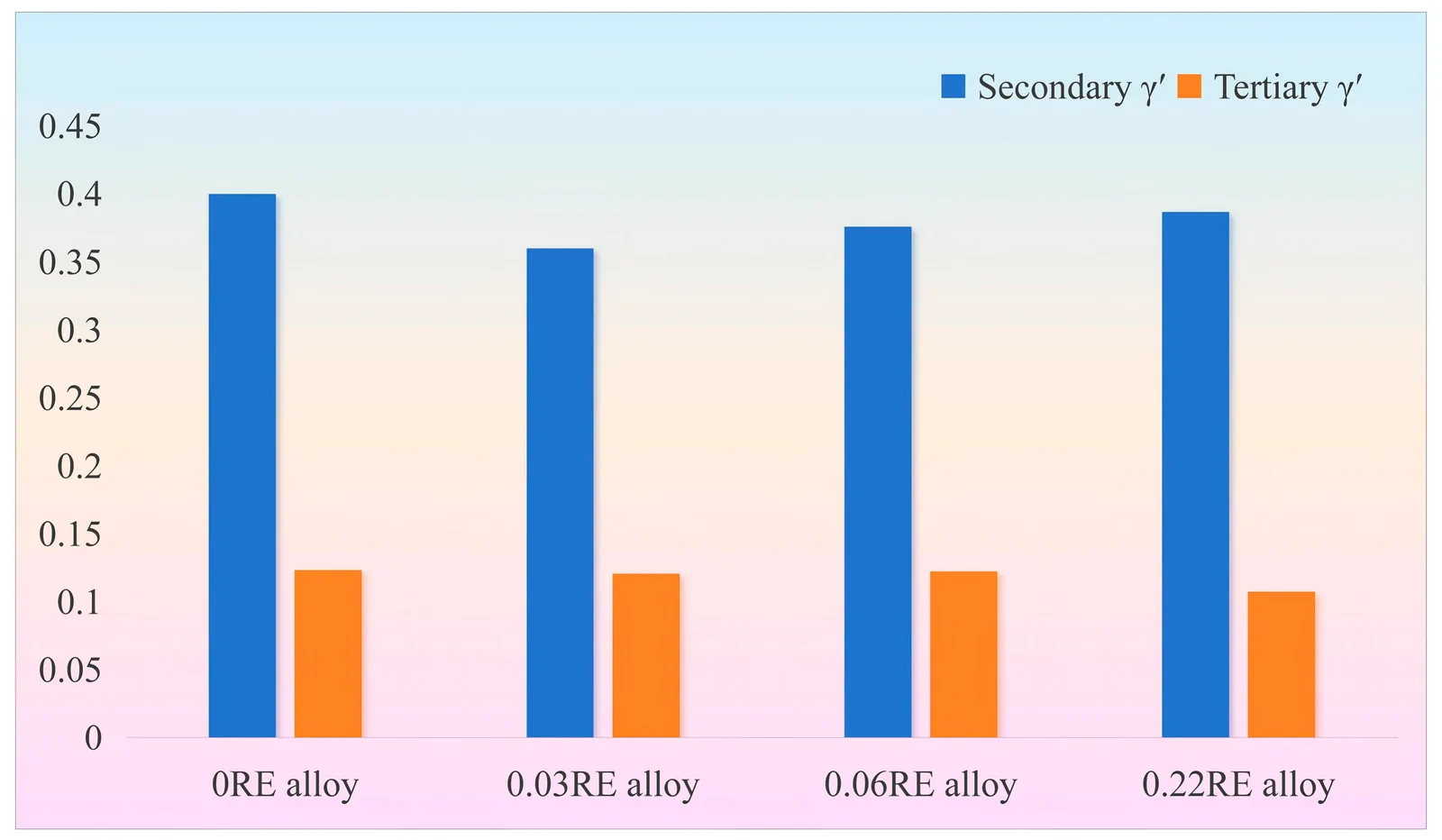
Average size of secondary γ′ phases and tertiary γ′ phases in different GH4079-based test alloys.

Figure 3 shows the morphologies of the γ′ phases in different GH4079-based test alloys. Since the previous heat treatment process had completely dissolved the primary γ′ phase, the main precipitates in the matrix were composed of secondary γ′ phases and tertiary γ′ phases. The large secondary γ’ phase exhibited a disk-like pattern, while the small tertiary γ’ phase was distributed in a granular form. Figure 4 presents the average sizes of the secondary γ′ phases and tertiary γ′ phases. The size of the secondary γ’ phase first decreased and then increased with the variation in RE. Furthermore, RE had no notable effect on the size of the tertiary γ’ phase.

**Figure 5:**
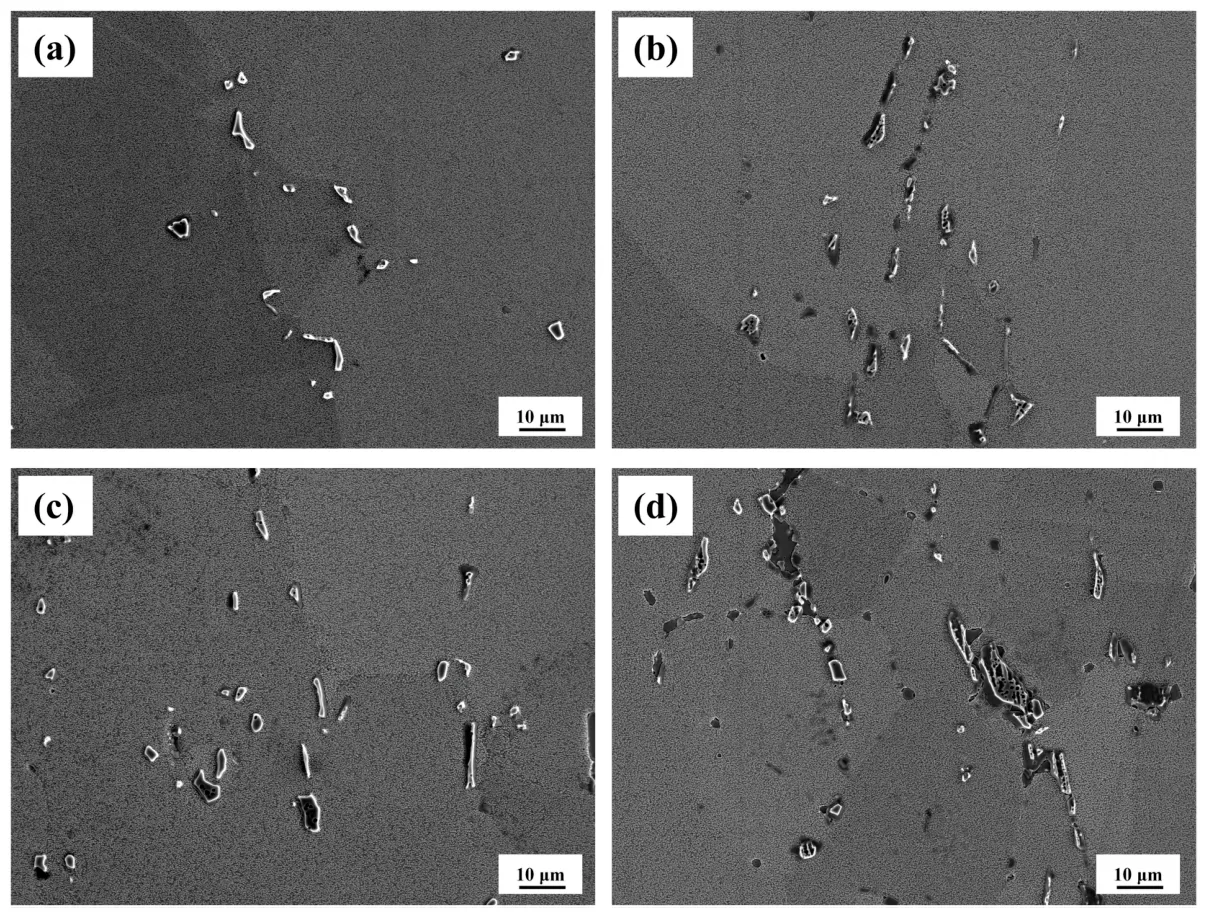
Precipitates in different GH4079-based test alloys: (**a**) 0RE alloy; (**b**) 0.03RE alloy; (**c**) 0.06RE alloy; (**d**) 0.22RE alloy.

Figure 5 displays the morphologies and distributions of precipitates in the four test alloys. Significant differences were observed in the distribution of precipitates, which were found both within grains and at grain boundaries. This indicated that the size and amount of precipitates increased with the addition of RE. The precipitates developed from small irregular blocks with an average size of approximately 3–5 μm in the 0RE alloy to large strip-like shapes with an average size of more than 10 μm in the 0.22RE alloy. 

**Figure 6:**
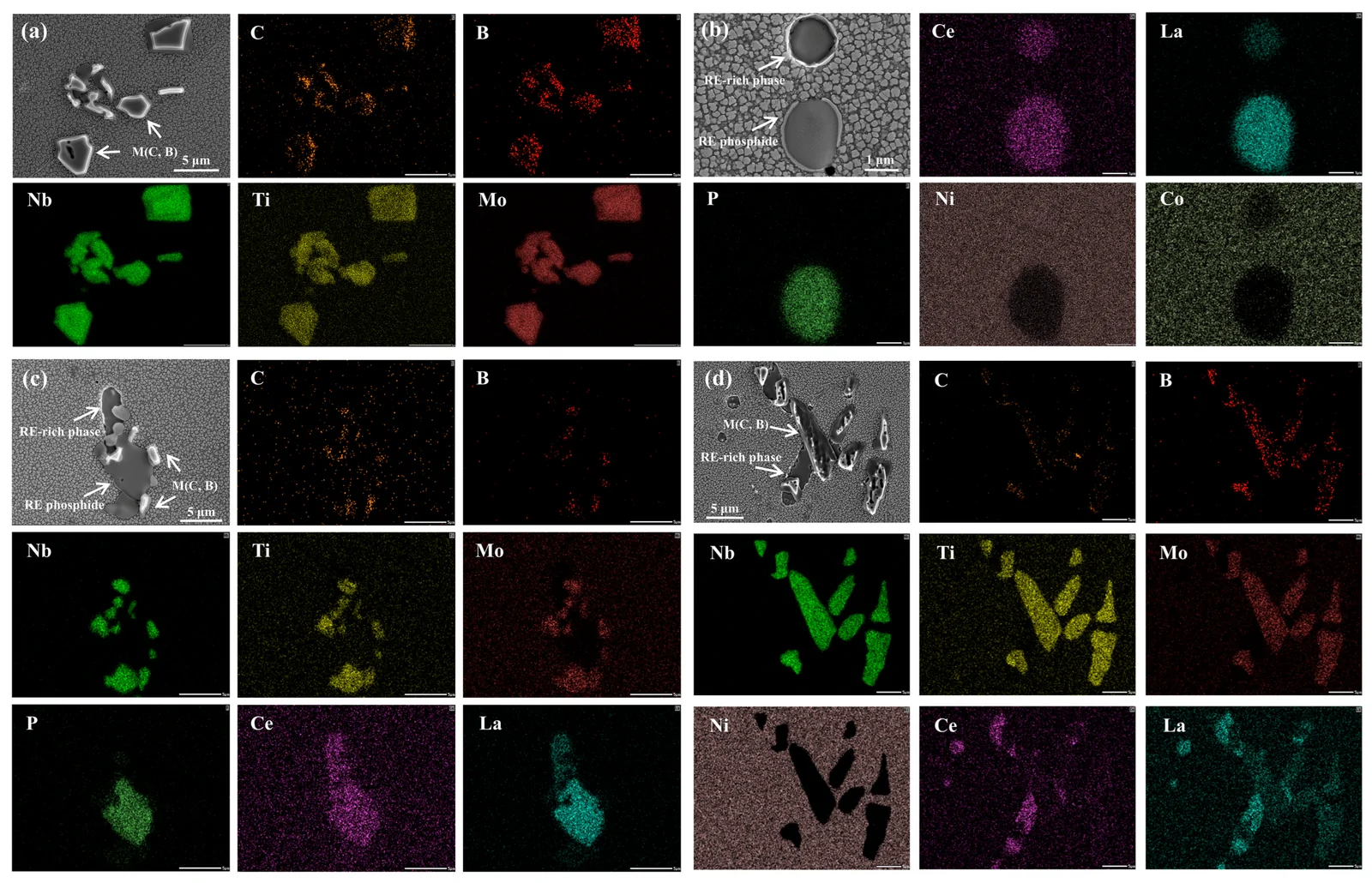
Morphology and element distribution of precipitates: (**a**) M(C,B) in 0RE alloy; (**b**) RE-rich phase and RE phosphide in 0.03RE alloy; (**c**) composite precipitate of M(C,B) and RE-rich phase, and RE phosphide in 0.06RE alloy; (**d**) co-precipitation of M(C,B) and RE-rich phase in 0.22RE alloy. The scale bar in each morphology panel applies to its corresponding element distribution panels.

The morphologies and element distributions of precipitates are shown in Figure 6. After the addition of RE elements, the transformation characteristics of the precipitates became significantly distinct. The precipitates changed from carbon/boron compounds rich in Nb, Ti, and Mo in the 0RE alloy to the formation of small RE phosphides and an RE-rich phase after adding 0.03% RE to the GH4079 alloy. As the RE content continued to increase to 0.06%, large RE-rich phases and RE phosphides coexisted with the carbon/boron compounds. The carbon/boron compounds seemed to act as the nucleation particles for the RE phase. When the content of RE increased to 0.22%, a large amount of the coexisting phases of carbon/boron compounds and RE phases was present in the matrix, and the size and amount of the precipitates significantly increased.

### 3.2. Stress Rupture Property

**Figure 7:**
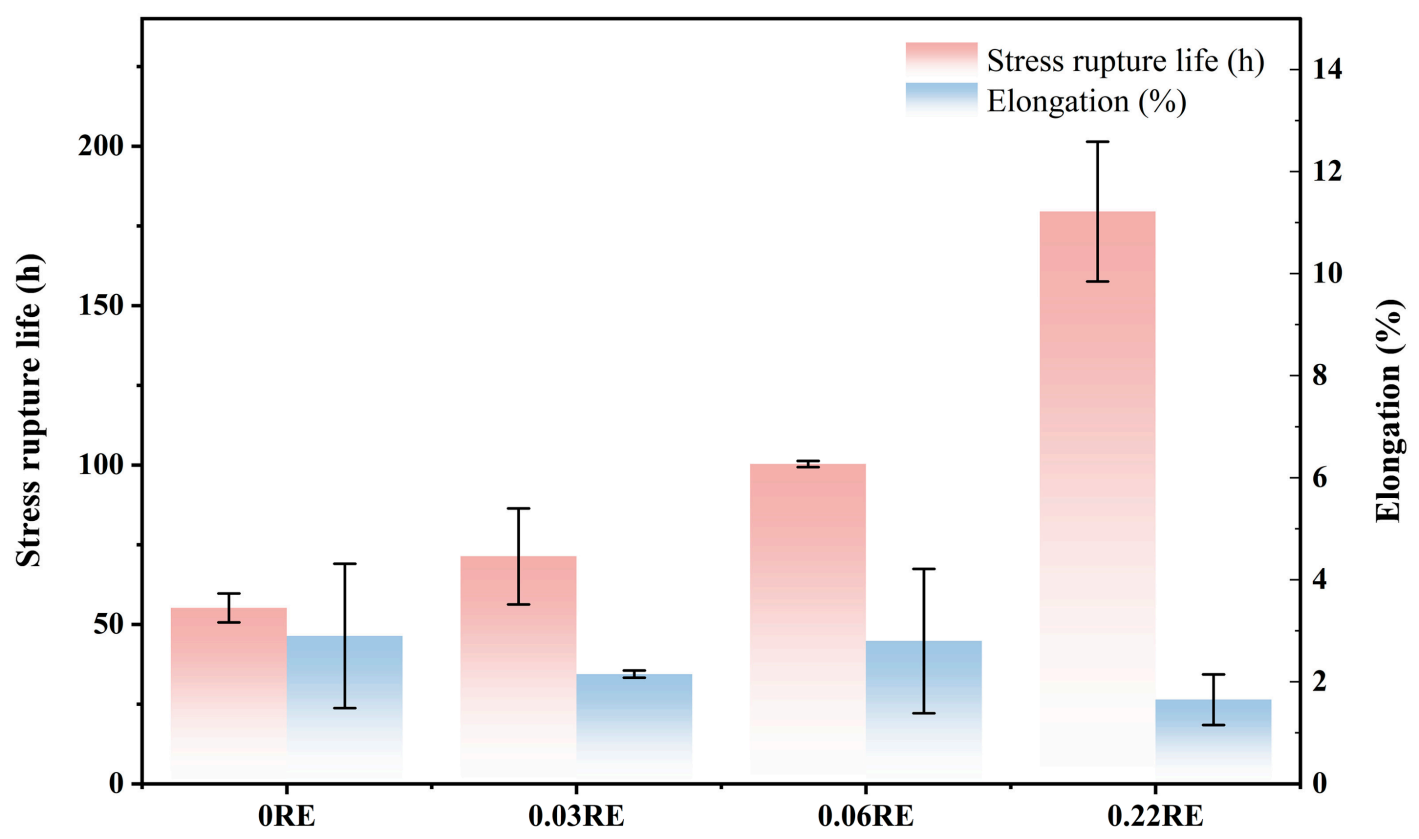
Stress rupture property of GH4079-based alloys.

Figure 7 shows the variation in the stress rupture properties of GH4079-based test alloys with the content of RE. The results clearly showed that the addition of RE increased the stress rupture life from 55.2 h (0RE alloy) to 179.3 h (0.22RE alloy), indicating that RE has a significant enhancing effect on the durability of the GH4079 alloy. 

**Figure 8:**
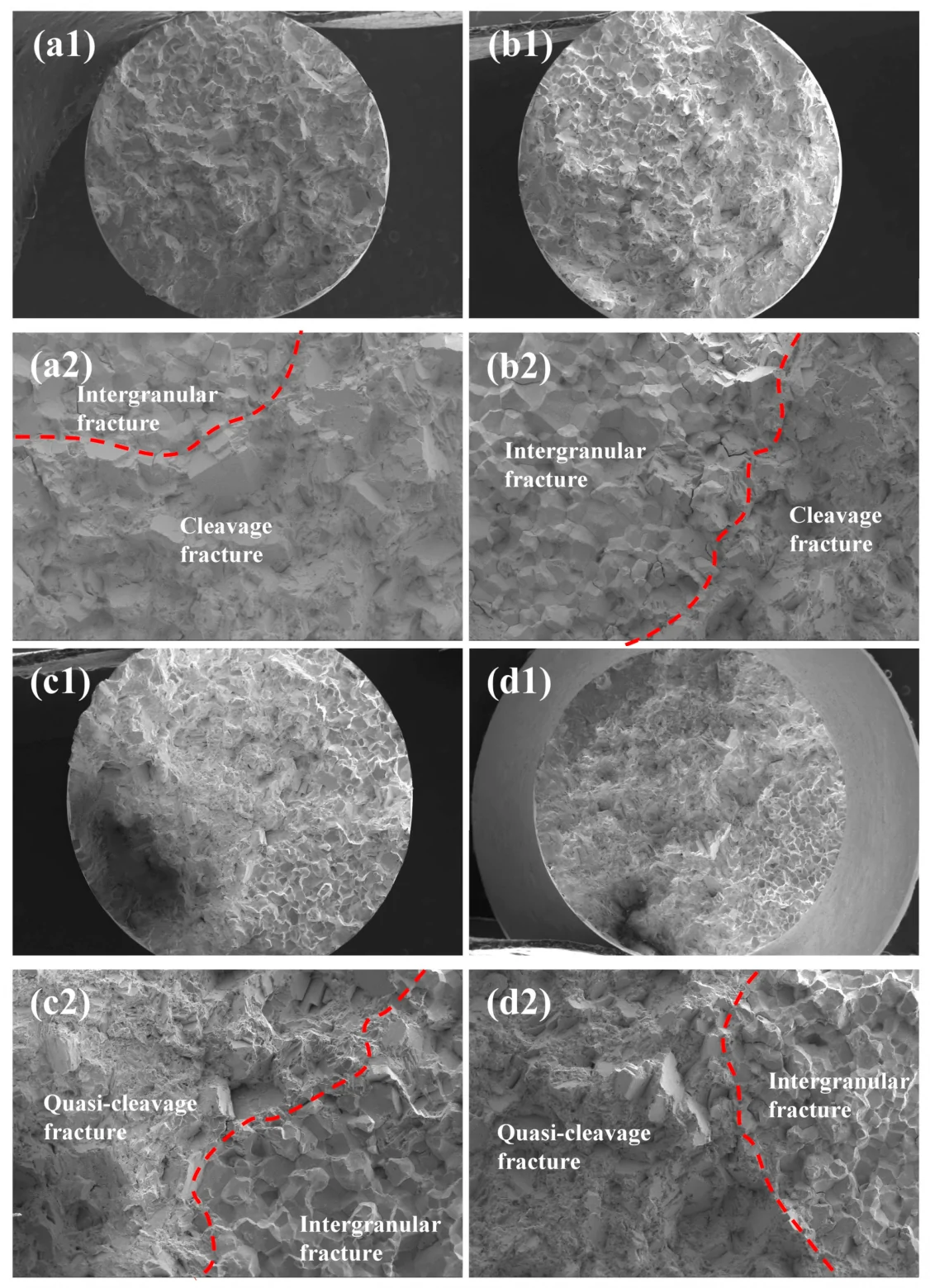
Fracture morphology of stress rupture samples: (**a1**,**a2**) 0RE alloy; (**b1**,**b2**) 0.03RE alloy; (**c1**,**c2**) 0.06RE alloy; (**d1**,**d2**) 0.22RE alloy.

The fracture surface morphologies of the tested stress rupture samples are shown in Figure 8. The surfaces of the 0RE alloy and the 0.03RE alloy presented typical brittle fracture characteristics, which were composed of a rock-candy pattern and cleavage planes with distinct boundary lines, as shown in Figure 8(a2,b2). The failure mode was typically an intergranular mixed cleavage fracture, indicating that the alloys have poor toughness and low grain-boundary strength. As the content of RE increased, the fracture surfaces exhibited the morphology of a quasi-plane slip fracture in the 0.06RE alloy and the 0.22RE alloy, as shown in Figure 8(c1,d1). Besides the small pseudo-planes, micropores and tearing edges also appeared on the fracture surfaces, indicating a transformation from a purely brittle fracture to a mixed-type fracture mode. The addition of RE may simultaneously enhance both the intragranular strength and grain-boundary strength, thus improving the stress rupture of the GH4079-based alloys.

## 4. Discussion

### 4.1. Effect of RE on γ′ Phase and Precipitates

**Figure 9:**
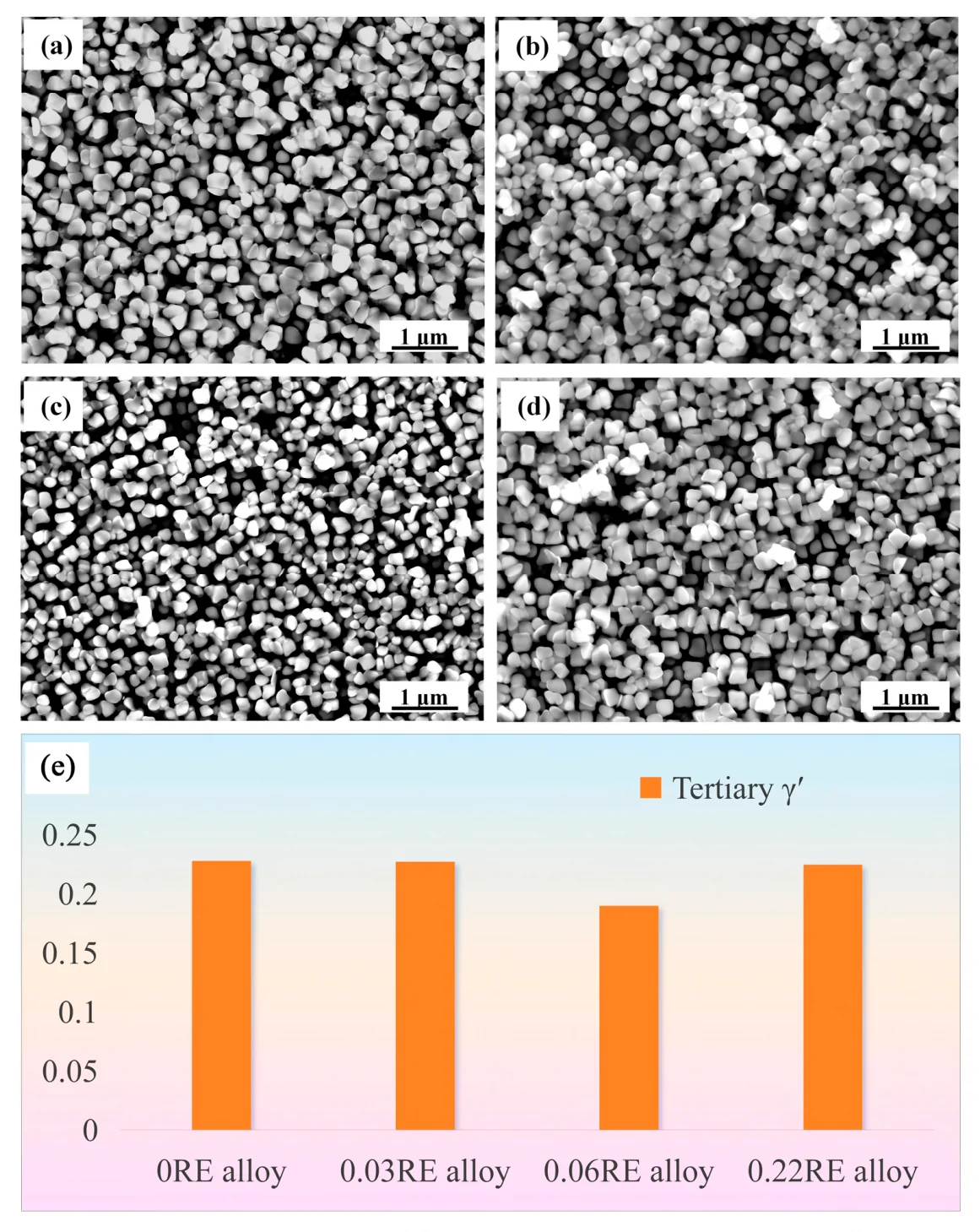
SEM images of γ′ phases after stress rupture tests: (**a**) 0RE alloy; (**b**) 0.03RE alloy; (**c**) 0.06RE alloy; (**d**) 0.22RE alloy and (**e**) calculated average size of γ′ phases.

With the addition of RE elements, the difference in the γ′ phase after initial forging and heat treatments was not obvious, as shown in Figure 3. However, the morphology, size and distribution of the γ′ phase after high-temperature stress rupture tests had significant changes, as shown in Figure 9. The large, block-like or butterfly-shaped secondary γ′ phase was completely redissolved, while the small-sized tertiary γ′ phase grew. Although 650 °C is still far from the equilibrium redissolution temperature of the γ′ phase, and the γ′ phase is unlikely to be redissolved under the sole effect of temperature, local stress-induced redissolution or stress-induced decomposition of the γ′ phase may occur under the combined effects of stress and temperature [31,32,33]. Therefore, it is entirely possible for the secondary γ′ phase to be redissolved into the matrix.

Generally, the misfit degree affects the morphology of the γ′ phase [34,35,36]. The square γ′ phase has a phase interface of the dislocation type or partial dislocation type, while the spherical γ′ phase has a coherent type interface with the γ matrix. The coherent stress increases with the increase in the misfit degree. Therefore, the γ′ phase is spherical when the misfit degree is small, and becomes square when the misfit degree is large. After adding RE elements, the γ′ phase in the alloy is significantly inhibited from growing, especially in the 0.06RE alloy. Previous studies have shown that RE elements accumulate at the interface of the γ/γ′ phases, which reduces the interfacial energy of the precipitated phase, slows down the diffusion rate of elements required for the growth of the γ′ phase, and ultimately inhibits the growth of the γ′ phase during the high-temperature stress rupture process [27,37,38]. The reason why the γ′ phase in the 0.22RE alloy grows is that the longer duration of the stress rupture test has coarsened the γ′ phase, while its size is still significantly smaller than that of the γ′ phase in the 0RE alloy. Combined with the results of this paper and existing studies, the inhibitory effect of RE elements on the growth of the γ′ phase is confirmed.

**Figure 10:**
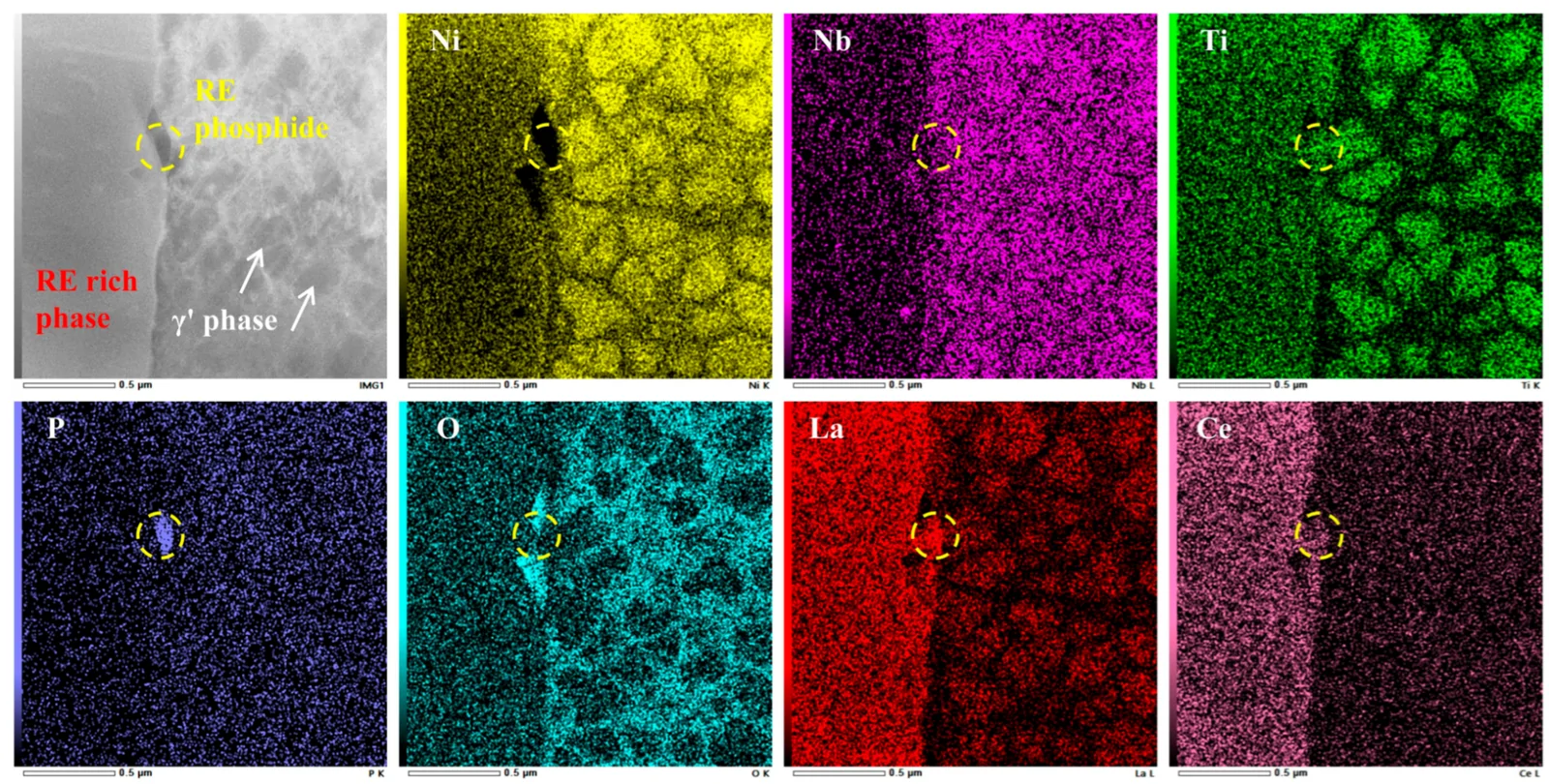
Morphology and element distribution of precipitates (RE-rich phase, RE phosphide, γ’ phase) by TEM.

It is well known that La and Ce have larger atomic radii than Ni. The supersaturated solidification RE atoms prevent the solubility of elements such as Ti, Nb, and Mo, and cause them to precipitate in the form of carbides. As the content of RE increased, the size of precipitated carbides or borides became larger and their quantity increased, as shown in Figure 6. Excess RE elements also precipitated in the form of an RE-rich phase or RE phosphides, along with the carbon/boron compounds. At the same time, the addition of La and Ce also altered the distribution coefficients of other elements in the γ and γ’ phases, reducing the content of other atoms with larger radii such as Ti, Nb, and Mo in the γ matrix and allowing the excluded atoms and some RE atoms to dissolve into the γ’ phase. Figure 10 shows the distribution of the precipitated phase elements by means of TEM. It can be seen that the distributions of La and Ti were highly consistent in the same location, indicating that La probably participated in the formation of the γ’ phase which was mainly Ni_3_(Al, Ti, Nb). Then Nb and Ti were excluded from the γ’ phase by La, and finally precipitated in the form of carbides, which explained why the amount of carbides in the 0.22RE alloy was significantly increased. It is worth noting that although La was found to participate in the formation of the γ’ phase, almost no Ce was observed. It was probably due to the difference in the atomic configurations of La and Ce. The electron activity of La is higher than that of Ce, achieving a small amount of Al, Ti and Nb substitution by occupying the vertices of the sublattice [17,39]. Therefore, in addition to affecting the size of the γ’ phase, RE also has a significant impact on the elemental composition of the γ’ phase.

**Figure 11:**
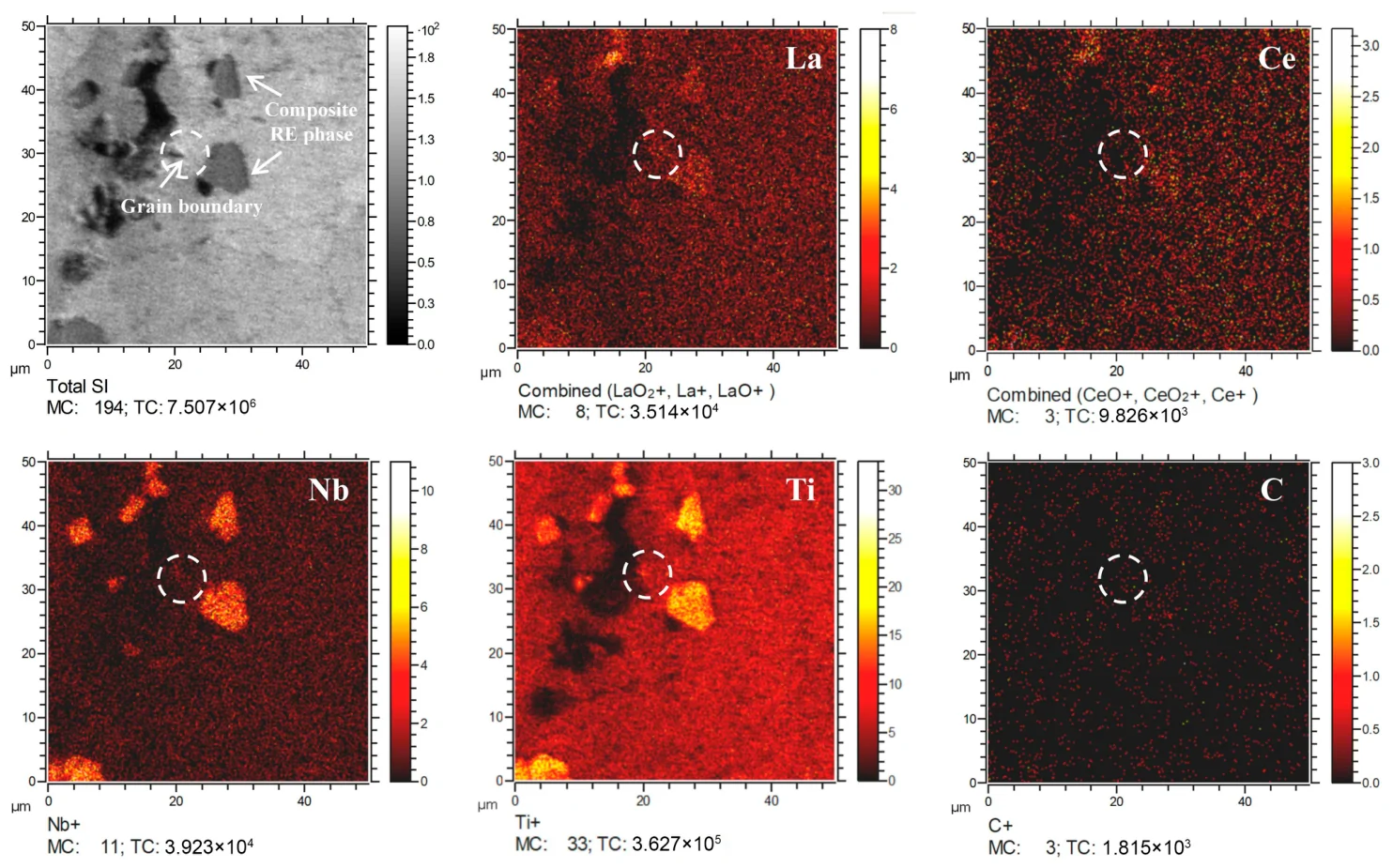
Distribution of La and Ce in 0.22% alloy characterized by SIMS.

The results of SIMS also demonstrated that the distributions of La and Ti were almost completely overlapping. Apart from the precipitated phase, the segregation at the grain boundaries was also basically consistent, as indicated by the white circle in Figure 11. However, the distribution of Ce was mostly in the RE-rich phase, and no obvious segregation was found at the grain boundaries, which showed a significant difference from La. The main reason is probably that the atomic radius of La is 188 pm, while that of Ce is 183 pm. The distortion energy of La in the matrix is higher, and the thermodynamic driving force for relaxation to reduce the distortion energy at the grain boundaries is greater. In addition, the La grain-boundary segregation energy is lower than that of Ce, making it easier for spontaneous grain-boundary segregation to occur [40,41].

### 4.2. Effect of RE on Grain Boundary

**Figure 12:**
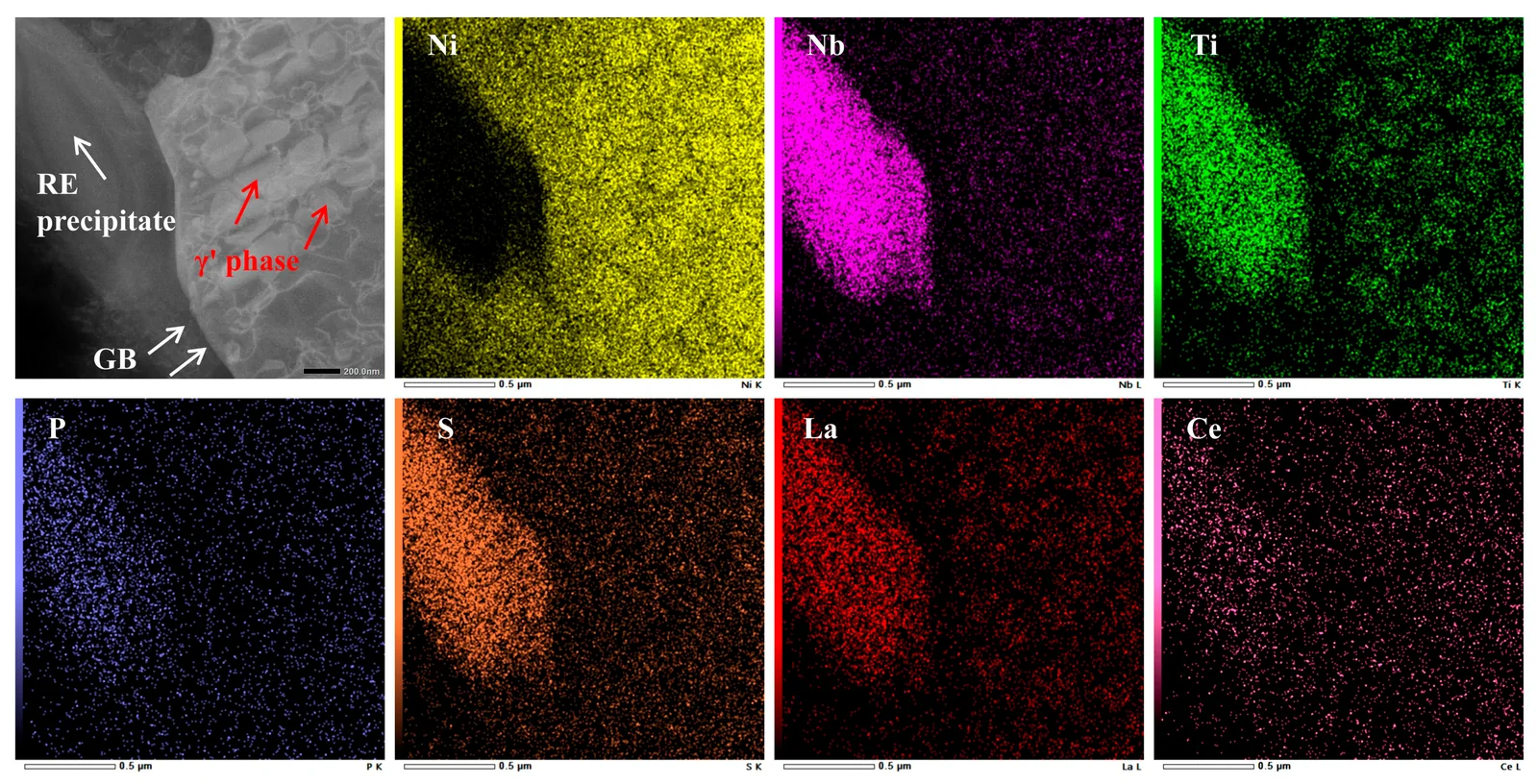
Precipitated phases and element distribution along grain boundaries by TEM.

In addition to having an impact on the precipitated phases, the segregation behavior of RE elements also has a significant effect on the grain boundaries [12,42,43]. It is well known that impurity elements such as P and S are often enriched at the grain boundaries, thereby weakening the strength of the grain boundaries [44]. Previous studies have confirmed that the addition of La and Ce can reduce the segregation of impurity elements at grain boundaries by forming compounds such as CeS and La_2_O_2_S, or by segregating at grain boundaries and occupying grain-boundary vacancies [45,46]. The beneficial effects of RE elements mainly lie in decreasing the segregation of P and S at the grain boundaries, which is achieved through the following two mechanisms. On the one hand, the segregation of the RE elements (demonstrated in Figure 11) occupied the vacancies at the grain boundaries, thus reducing the segregation of harmful elements to the grain boundaries and enhancing the interface bonding strength. On the other hand, numerous small precipitated phases containing RE elements were found at the interface, as shown in Figure 12. No enrichment of S and P elements was observed at the grain boundaries. When RE elements reacted with P and S to form precipitates along the grain boundaries, the contents of S and P at grain boundaries were reduced, thereby purifying the grain boundaries and avoiding the negative effects that weaken the grain-boundary bonding force. 

### 4.3. Effect of RE on Stress Rupture Property

**Figure 13:**
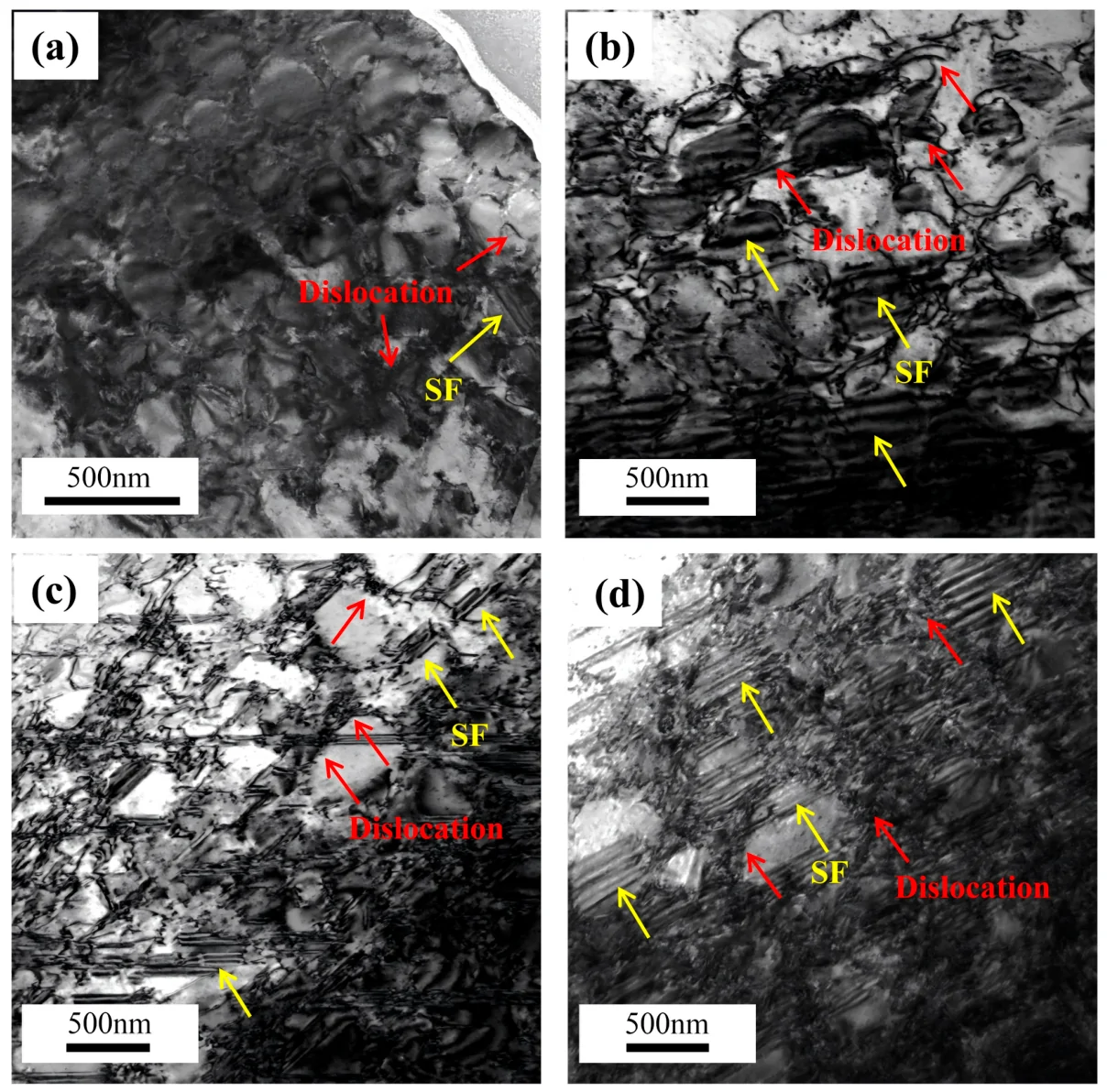
Dislocation patterns of in different GH4079-based test alloys: (**a**) 0RE alloy; (**b**) 0.03RE alloy; (**c**) 0.06RE alloy; (**d**) 0.22RE alloy.

The reasons for the improvement in the stress rupture life of the GH4079-based alloy with RE additions lie in the combined strengthening effects of intracrystalline γ′ phase strengthening and grain-boundary strengthening. First, RE elements participate in the formation of the γ′ phase and inhibit its growth, making it stay in the form of small spherical particles and resulting in a good strengthening effect [11,17,47]. This was verified by the transformation of dislocation patterns after stress rupture testing, as shown in Figure 13. In the samples of the 0RE alloy and the 0.03RE alloy, there were few stacking faults (SF) inside the γ′ phase, and the dislocations cut through γ′ phase, indicating that the γ′ phase was relatively soft and the strengthening effect was weak. As the content of RE elements increased, a large number of SFs and dislocations appeared in the matrix. Since RE elements had participated in the formation of the γ′ phase, the strength of γ′ phase was enhanced. Therefore, when dislocations encountered the γ′ phase, they could not pass through the γ′ phase but could only bypass it and form dislocation loops. This indicates that the strength of the γ′ phase has increased and the strengthening effect has been enhanced.

**Figure 14:**
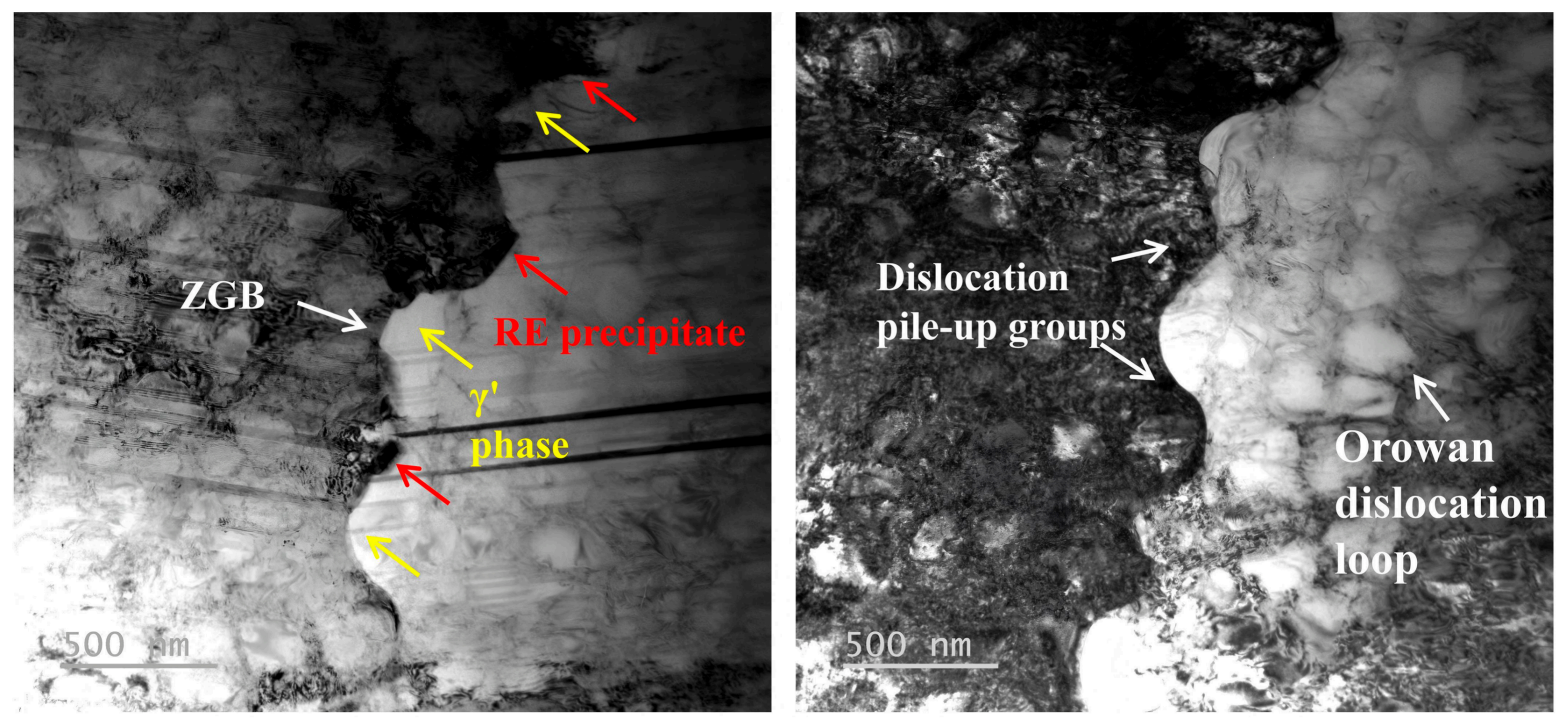
The formation of zigzag grain boundary (ZGB) and patterns of dislocation.

Furthermore, the strengthening effect of RE elements on grain boundaries is also crucial for the improvement of stress rupture life. As mentioned above, the segregation of RE elements at grain boundaries can play a role in purifying the grain boundaries and enhancing the bonding force, contributing to the increase in stress rupture life. Moreover, the small RE-rich precipitates on the grain boundaries also work together with the γ′ phase to exert a pinning effect on dislocations, as shown in Figure 14. Under the combined effect of fine RE-containing precipitates and the γ′ phase that were uniformly distributed at the grain boundaries, a zigzag grain boundary (ZGB) was formed. ZGB can increase the area of grain boundaries and help disperse the stress concentration along the grain boundaries, which may delay the initiation and propagation of intergranular cracks during the long-term, high-temperature stress loading process, thereby contributing to the improvement of the stress rupture life of the alloy [48]. 

The strengthening effect of RE elements at the grain boundaries is probably higher than that within the matrix, because the precipitation of associated carbides becomes the nucleation sites for fracture, thus resulting in the formation of dimples and initiating the transition to ductile fracture. The fine and uniformly distributed γ′ phase can better hinder the movement of dislocations, which provides a foundation for the improvement of the alloy’s stress rupture performance.

## 5. Conclusions

In this work, the influences of different lanthanum (La) and cerium (Ce) additions on the initial microstructure and stress rupture properties of the GH4079 superalloy were systematically investigated, and the strengthening mechanisms of La and Ce in the alloy were clarified. The main conclusions are summarized as follows.

1. La and Ce can effectively inhibit the excessive growth of the γ′ phase, maintaining a fine and uniform distribution of γ′ phase particles. As the content of RE elements increased, the amount and size of the precipitated RE-rich phases and carbides significantly increased.
2. La and Ce significantly improved the stress rupture life of GH4079-based alloys at 650 °C/882 MPa, and the alloy with a 0.22 wt.% RE addition exhibited the best comprehensive stress rupture properties. The stress rupture life of the 0.22RE alloy reached 179.3 h, compared with 55.2 h for the RE-free alloy, representing a more than threefold increase.
3. The strengthening mechanisms of La and Ce in GH4079-based alloys were reflected in two aspects: intracrystalline and grain-boundary strengthening. On the one hand, RE elements participated in the formation of the γ′ phase, thus improving the strength of the γ′ phase and enhancing the dislocation hindering effect. On the other hand, RE elements purified the grain boundaries by reducing the segregation of the harmful elements P and S and formed fine RE-containing precipitates to induce the formation of zigzag grain boundaries, which relieved stress concentration along the grain boundaries and delayed the propagation of intergranular cracks, ultimately improving the stress rupture performance of the alloy.

## Figures and Tables

**Table 1:** Chemical compositions of GH4079 alloys (wt.%).

Sample	Cr	Co	Mo	Al	Nb	Ti	Fe	V	W	C	B	La	Ce	Ni
0RE	11.06	14.08	4.33	2.94	2.67	2.60	0.085	0.60	2.40	0.041	0.0099	-	-	Bal.
0.03RE	10.97	14.06	4.33	3.00	2.67	2.58	0.079	0.60	2.40	0.042	0.0095	0.025	0.005	Bal.
0.06RE	10.90	14.11	4.34	3.04	2.65	2.59	0.14	0.60	2.42	0.039	0.0093	0.050	0.010	Bal.
0.22RE	10.89	14.06	4.31	3.27	2.63	2.59	0.059	0.60	2.42	0.043	0.0097	0.150	0.070	Bal.

## Data Availability

The original contributions presented in this study are included in the article. Further inquiries can be directed to the corresponding authors.

## References

[1] Lu T., Liu X., Zhang W., Tian Q. (2024). Microstructure Evolution and Fracture Characteristics of GH4079 Superalloy during High-Temperature Tensile Process. Mater. Sci. Eng. A.

[2] Zhang X., Tao C., Zhou G., Zhang H., Zhang S., Ma X., Gan B., Chen L. (2025). The Contribution of γ’ Phase Transition Induced (PI-CDRX)—CDRX-DDRX Alternating Cycling Behavior to the Hot Formability of GH4079 Alloy. Mater. Charact..

[3] Osada T., Nagashima N., Gu Y., Yuan Y., Yokokawa T., Harada H. (2011). Factors Contributing to the Strength of a Polycrystalline Nickel–Cobalt Base Superalloy. Scr. Mater..

[4] Zhong L.Q., Liang Y.L., Hu H. (2017). Study on Plastic Deformation Characteristics of Shot Peening of Ni-Based Superalloy GH4079. IOP Conf. Ser. Mater. Sci. Eng..

[5] Zhang W., Liu X., Lu T., Liu Y., Guo Y., Qin H., Tian Q. (2024). Microstructural Evolution of GH4079 Superalloy during Hot Deformation and Heat Treatment. J. Mater. Res. Technol..

[6] Ying W., Hou J., Jiang S., Wang J. (2025). A Comparison of the Hot Deformation Behavior and Constitutive Model of the GH4079 Alloy. Crystals.

[7] Zhang X., Zhou G., Zhang H., Zhang S., Ma X., Gan B., Chen L. (2024). Study of Processing Map of GH4079 Alloy Based on Different Instability Criteria. JOM.

[8] Golmakaniyoon S., Mahmudi R. (2011). Comparison of the Effects of La- and Ce-Rich Rare Earth Additions on the Microstructure, Creep Resistance, and High-Temperature Mechanical Properties of Mg–6Zn–3Cu Cast Alloy. Mater. Sci. Eng. A.

[9] Chen W., Gong C., Jiang P., Gan L., Ren Y., Li C., Chen J., Qiu W. (2025). Enhancing Corrosion Resistance of Mg-Alloys by Regulating Precipitates at Grain Boundaries Using Rare Earth Oxides. J. Mater. Sci. Technol..

[10] Cao S., Yang Y., Chen B., Liu K., Ma Y., Ding L., Shi J. (2021). Influence of Yttrium on Purification and Carbide Precipitation of Superalloy K4169. J. Mater. Sci. Technol..

[11] Bian W., Zhang H., Zhang X., Gao M., Li J., Li Q., Cui Y., Zhang H. (2019). Comprehensive Influence of Y on K417 Superalloy: Purification, Interactions among the Alloy Elements and High Temperature Properties. Mater. Sci. Eng. A.

[12] Tian Q., Huang S., Qin H., Duan R., Wang C., Lian X. (2024). Synergistic Effects of Boron and Rare Earth Elements on the Microstructure and Stress Rupture Properties in a Ni-Based Superalloy. Materials.

[13] Da Silveira R.M.S., Guimarães A.V., De Almeida L.H., Paula A.D.S., Farina A.B., Malet L., Dille J., Araujo L.S. (2025). Effect of Yttrium Addition on Creep Behavior of Wrought Nickel Alloy 718. Mater. Sci. Eng. A.

[14] Hu J., Chen M., Huang S., Tian Q., Wang C., Yu H., Chen Y., Xu W. (2026). Phosphorus-Rare Earth Co-Precipitations and Twinning Render Enhancement of High Temperature Plasticity in Ni-Based Superalloy. J. Mater. Sci. Technol..

[15] Zhou P.J., Yu J.J., Sun X.F., Guan H.R., Hu Z.Q. (2007). Role of Yttrium in the Microstructure and Mechanical Properties of a Boron-Modified Nickel-Based Superalloy. Scr. Mater..

[16] Palleda T.N., Chowdhury H.T., Banoth S., Murakami H., Kakehi K. (2024). Effects of Yttrium Content on Solidification, Microstructure, and Mechanical Properties of Laser Powder Bed Fused IN718 Superalloy. J. Alloys Compd..

[17] Rong L., Wang M., Xing W., Hao X., Ma Y. (2023). Effects of Cerium Addition on the Microstructure and Stress Rupture Properties of a New Nickel-Based Cast Superalloy. J. Mater. Sci. Technol..

[18] Guan K., Huang Z., Cui R., Qin J. (2019). Effects of Yttrium on Microstructure and Mechanical Properties of a Directionally Solidified Single Crystal Superalloy. Mater. Sci. Eng. A.

[19] Song X., Wang L., Liu Y., Ma H.P. (2012). Study on the Mechanical Performance of a Ni-Based Superalloy with Trace Rare Earth Element La Additions. Adv. Mater. Res..

[20] Mao W., Zhang L., Niu Z., Liu H., Yu Y., Zhang J., Zhang S., Yan W. (2026). Effect of Lanthanum on Inclusions and Solidification Structure of Alloy 800H. Ironmak. Steelmak. Process. Prod. Appl..

[21] Song X., Wang L., Liu Y., Ma H. (2011). Effects of Temperature and Rare Earth Content on Oxidation Resistance of Ni-Based Superalloy. Prog. Nat. Sci. Mater. Int..

[22] Li X., Ou M., Wang M., Zhang L., Ma Y., Liu K. (2021). Effect of Boron Addition on the Microstructure and Mechanical Properties of K4750 Nickel-Based Superalloy. J. Mater. Sci. Technol..

[23] Antonov S., Després A., Mayer C., Martin G., Kontis P. (2023). Boron Trapping at Dislocations in an Additively Manufactured Polycrystalline Superalloy. Materialia.

[24] Ou M., Ma Y., Ge H., Chen B., Zheng S., Liu K. (2018). Effect of Long-Term Aging on the Microstructure, Stress Rupture Properties and Deformation Mechanisms of a New Cast Nickel Base Superalloy. Mater. Sci. Eng. A.

[25] Zhang P., Yuan Y., Zhong L., Gu Y.F., Yan J.B., Lu J.T., Yang Z. (2021). Microstructural Stability and Tensile Properties of a New Γ′-Hardened Ni-Fe-Base Superalloy. Materialia.

[26] Huang Y., Zhang R., Zhou Z., Yan J., Yuan Y., Gu Y., Cui C., Zhou Y., Sun X. (2022). Effect of Long-Term Aging on Microstructural Stability and Tensile Deformation of a Fe–Ni-Based Superalloy. Mater. Sci. Eng. A.

[27] Li J.G., Wang N., Liu J.D., Xu W. (2024). Influence of Rare Earth Elements (Y, La and Ce) on the Mechanical Properties and Oxidation Resistance of Nickel-Based Superalloys: A Critical Review. J. Mater. Sci. Technol..

[28] (2025). Superalloys and Intermetallic Compounds—Designation and Chemical Composition.

[29] (2024). Metallic Materials—Uniaxial Creep Testing Method in Tension.

[30] (2001). Center Holes.

[31] Wang J., Huang H., Xin D., Qu J., Zhang H., Ruan J., Zhou X., Zhang S., Jiang L. (2024). The Evolution Behavior and Mechanism of γ’ Particles during Hot Deformation in a New P/M Nickel-Based Superalloy. Mater. Charact..

[32] Park K., Davies J., Withey P. (2025). Microstructural Investigation of Stress-Induced Degradation of Gamma and Gamma Prime Phases on the Surface of the Aerofoil of Nickel-Based Single Crystal Superalloy Turbine Blades. Crystals.

[33] Yang Q.-M., Lin Y.C., Liu G., Chen Z.-J., Qiu Y.-L., Wu G.-C., Zhu J.-C. (2023). Morphological Evolution and Dissolution Mechanisms of Γ′ Phase in a Novel HIPed P/M Superalloy in Hot Deformation Process. J. Alloys Compd..

[34] Yamaguchi Y., Tajima R., Terada Y. (2020). Morphology Evolution of Γ′ Precipitates for Wrought Ni-Based Superalloys. Mater. Trans..

[35] Van Sluytman J.S., Pollock T.M. (2012). Optimal Precipitate Shapes in Nickel-Base γ–Γ′ Alloys. Acta Mater..

[36] Zhang P., Yuan Y., Yan J.B., Wang J.C., Song X.L., Yang G.X. (2018). Morphological Evolution of Γ′ Precipitates in Superalloy M4706 during Thermal Aging. Mater. Lett..

[37] Wang N., Liu J., Wang X., Xu W., Li J. (2026). Inhibiting Dislocation Recovery via Yttrium-Induced Diffusion Barriers: A Novel Strategy for Creep Resistance in Ni-Based Single Crystal Superalloys. Scr. Mater..

[38] Shen J., Zhu X., Jiang H., Dong J. (2025). An Investigation on the Effect of Sc Microalloying on Creep Property Improvement in Γ′-Phase Strengthened Nickel-Based Superalloy. Mater. Sci. Eng. A.

[39] Cheng P. (2023). Lanthanides: Fundamentals and Applications.

[40] Liu Y., Zhi J., Lyu Z., Gu C., Diao W., Qu Z., Bao Y. (2025). Effect of Lanthanum-Cerium Rare Earth Elements on Steel at Atomic Scale: A Review. Metals.

[41] Cao J., Yang W., He X. (2019). Segregation Behavior and Embrittling Effect of Lanthanide La, Ce, Pr, and Nd at Σ3(111) Tilt Symmetric Grain Boundary in α-Fe*. Chin. Phys. B.

[42] Lin Q., Guo F., Zhu X. (2007). Behaviors of Lanthanum and Cerium on Grain Boundaries in Carbon Manganese Clean Steel. J. Rare Earths.

[43] Zhang S., Yu J., Li H., Jiang Z., Ren J., Feng H., Zhu H., Zhang B., Han P. (2025). Unveiling the Role of Cerium in Enhancing the Hot Ductility of Super Austenitic Stainless Steel S32654 at Different Temperatures. J. Mater. Sci. Technol..

[44] Wang L.-M., Lin Q., Yue L.-J., Liu L., Guo F., Wang F.-M. (2008). Study of Application of Rare Earth Elements in Advanced Low Alloy Steels. J. Alloys Compd..

[45] Cosandey F., Li D., Sczerzenie F., Tien J.K. (1983). The Effect of Cerium on High Temperature Tensile and Creep Behavior of a Superalloy. Metall. Trans. A.

[46] Song X., Wang L., Liu Y., Ma H. (2010). Precipitation Characteristics and La Effects on Precipitates of a New 22Cr-14W-2Mo Superalloy. Rare Met..

[47] Jin Y., Han P., Li C., Kou S. (2016). Effect of Rare Earth Elements (Y,Ce) on Microstructure of K418 Ni-Base Superalloy. J. Mater. Eng..

[48] Zhang W., Lu T., Zha J., Zhang Z., Li H., Liu X., Tian Q., Wang K. (2026). Influence of Fan-Shaped Microstructure and Serrated Grain Boundaries on the Intermediate-Temperature Tensile Properties of Superalloys. J. Alloys Compd..

